# Treatment of a Recurrent Periprosthetic Joint Infection with an Intramedullary Knee Arthrodesis System with Low-Amount Metallic Silver Coating

**DOI:** 10.7150/jbji.34484

**Published:** 2019-04-20

**Authors:** Volker Alt, Christian Heiss, Markus Rupp

**Affiliations:** 1 Department of Trauma Surgery, Regensburg University Medical Center, Germany; 2 Department of Trauma, Hand and Reconstructive Surgery, University Hospital Giessen-Marburg, Campus Giessen, Germany

**Keywords:** silver, periprosthetic joint infection, knee, arthrodesis, Staphylococcus

## Abstract

We present a case of a recurrent periprosthetic knee infection treated with an intramedullary arthrodesis system coated with low amounts of metallic silver. After a follow-up of 26 months, the patient remained infection free and no silver-related complications were detected. Silver serum concentrations remained below the detection limit of 2 ppb.

## Introduction

Recurrent or recalcitrant periprosthetic joint infection after revision total knee arthroplasty remains a difficult-to-treat complication in orthopaedic surgery that requires knee arthrodesis in some cases, particularly if the extensor mechanism is significantly damaged 1. There are different surgical techniques for knee arthrodesis, including intramedullary devices that can additionally address segmental bone defects 2, 3. Also, knee arthrodesis is related with high reinfection rates 4-8 and silver-coating with its broad antimicrobial effects has the potential to combat biofilm formation on implants 9 and silver has already been described in order to reduce this complication 2. This silver-technology (Modular Universal Tumor And Revision System (MUTARS^®^, Implantcast, Buxtehude, Germany) is achieved by galvanic deposition of elemental silver (with a percentage purity of 99.7%) onto the surface of the titanium-vanadium prostheses with a thickness of the coating of 15 µm (±5 µm) and another layer of gold of 0.2 µm thick to ensure sustained release of silver ions. This is associated with relatively total high amounts of silver of up to 2.89 g in tumor megaendoprostheses 10. Silver-specific complications, including argyria, which is local blue to bluish grey skin discoloration, and elevated silver concentrations have been reported after the use of this technology 10, 11. Another downside of this technology is that intramedullary parts of the device are not coated.

We here present a case of a recurrent periprosthetic knee infection successfully treated with an intramedullary knee arthrodesis system coated with an alternative silver technology that uses low amounts of metallic silver and allows for full coating of the implant including its intramedullary parts 12. This technology relies on elemental silver particles that are embedded in a SiOxCy plasma polymer layer. Further technical details can be derived from Khalilpour et al. (2011) 12. The coating has demonstrated good *in vitro* antimicrobial activity against *St. epidermidis*, methicillin-sensitive *St. aureus* and methicillin-resistant *St. aureus* (MRSA), and good *in vivo* biocompatibility in rabbits 12.

## Case report

**Figure 1:**
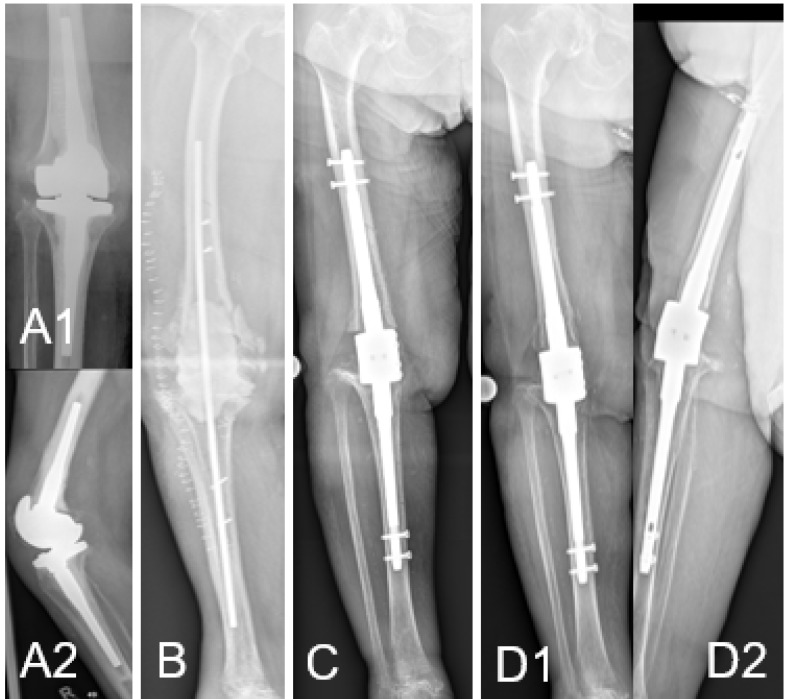
** (A)** Pre-operative X-ray with knee revision arthroplasty with cemented femoral and tibial stem anchorage without any obvious signs of loosening or osteolysis (A1: ap, A2: lateral). **(B)** After removal of the revision prosthesis intramedullary rods are placed in the femur and tibia with an antibiotic-loaded PMMA spacer to fill the large dead space as interim knee arthrodesis solution. Cortical windows were needed for complete bone cement removal, which were then fixed with screws. **(C)** Postoperative ap-view after modular knee arthrodesis with metallic silver coating and connection of the intramedullary femoral and tibial components with a spacer and locking bolts. Intramedullar rods were fixed cementless. **(D)** 26 months control after modular knee arthrodesis no implant loosening could be evidenced on both ap (D1) and lateral (D2) view.

An 86-year-old woman presented with persistent pain and swelling of her right knee 16 months after infection-related revision knee arthroplasty. A sinus tract was visible on the medial side of the distal thigh. Purulent discharge emptied on pressure. The elderly woman suffered from congestive heart failure, hypertension, aortic stenosis which had previously required aortic valve replacement surgery and had an ASA score of 3. The X-ray showed a knee revision arthroplasty with cemented femoral and tibial stem anchorage without any obvious signs of loosening or osteolysis (Fig. 1A).

A two-stage procedure as curative therapy strategy was planned and carried out for the patient. During the first intervention, a significant loss of the patellar tendon due to the infection and the previous interventions was found. After thorough debridement, titanium rods were coated with Copal^®^ cement (Heraeus, Wehrheim, Germany), to which was added vancomycin (40g Copal^®^ cement + 2g vancomycin powder) in a silicon tubing technique. The rods were placed in the medullary canal of the femur and tibia. Additionally, a cement spacer was placed in the dead space, which resulted from removal of the knee prosthesis and bone resection (Fig. 1B).

**Figure 2:**
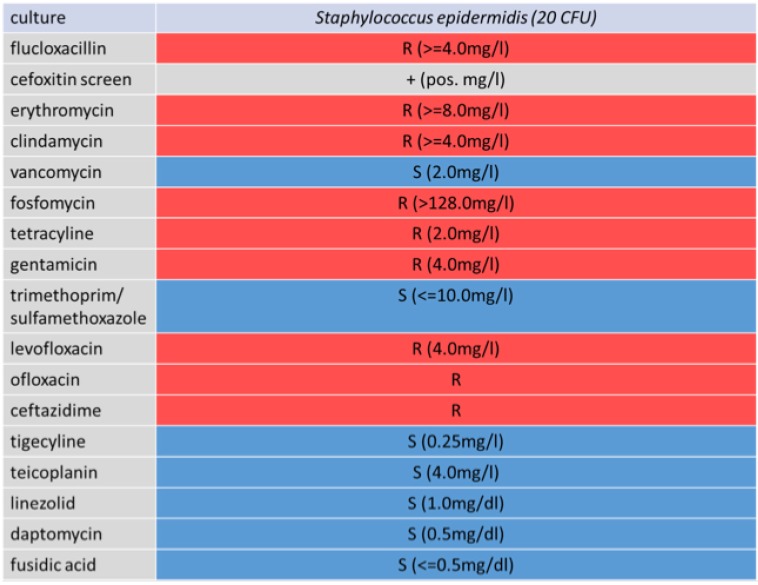
Antibiogram of the infection causative *St. epidermidis*.

Initial empiric antibiotic therapy with ampicillin /sulbactam 2000mg/1000mg three times a day was started, and changed as to vancomycin i.v. after the diagnosis of *S. epidermidis*, with a relatively high resistance profile, including resistance against methicillin, rifampicin and fluoroquinolones (Fig. 2).

On postoperative day 18, an unplanned revision surgery was necessary due to persistent wound secretions with repeated debridement and the antibiotic PMMA-spacer and cement-coated rods were changed. Biopsies taken during this intervention remained sterile.

After subsequent uneventful wound healing and improved laboratory parameters (leucocyte count, C-reactive protein levels) stage 2 surgical procedure was planned. Due to the loss of the patellar tendon with subsequent loss of active knee extension and a history of recurrent prosthetic joint infection, knee arthrodesis was advocated.

**Figure 3:**
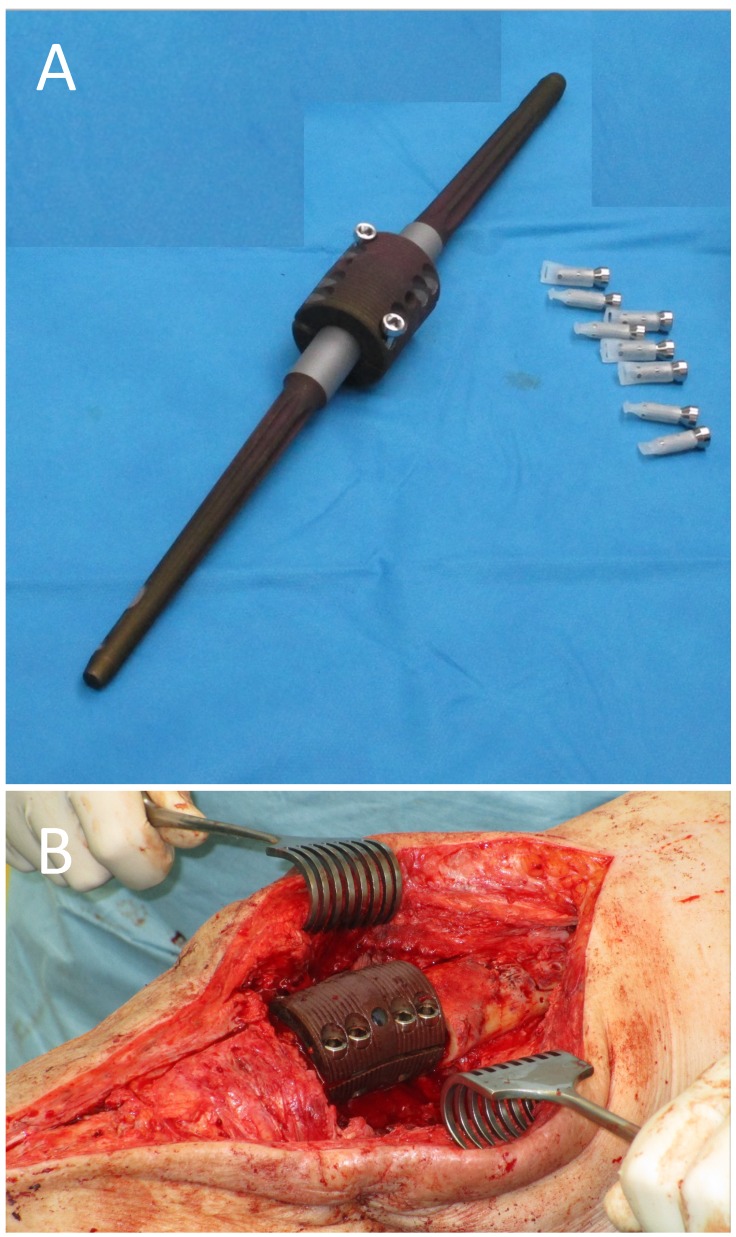
(**A**) Metallic silver-coated intramedullary knee arthrodesis system with locking bolts (uncoated) The Metallicsilver coated parts (intramedullary femoral, intramedullary tibial and spacer) appear black. (**B**) Intraoperative image after implantation of the silver-coated knee arthrodesis system with complete locking of the two spacer parts with 8 locking bolts (4 lateral, 4 medial (not visible)) between the intramedullary femoral component (right) and the intramedullary tibial component (left).

Pre-operative planning was done using an intramedullary knee arthrodesis system (OsteoBridge^®^, Merete GmbH, Berlin, Germany). Femoral and tibial locking intramedullary implants with a diameter of 16 mm for the femur and 14 mm for the millimeters with a length of 200 mm each were selected (Fig. 3A). Connection of these two intramedullary implants was planned with a central spacer consisting of two part with a length of 50 mm and a diameter of 40 mm each. The femoral and tibial intramedullary components as well as the central spacer connecting the femoral and tibial parts were pre-operatively coated with a 90 nm thick siloxane-microsilver coating (HyProtect™, Bio- Gate AG, Nürnberg, Germany) with a relatively low amount of silver (Fig. 3B). The silver concentration of the implant was 2.7 µg/cm^2^ with a total silver content of the implant of around 825 µg.

Knee arthrodesis surgery was carried out three months after the removal of the prosthesis and the silver coated arthrodesis device was implanted without any problems (Fig. 3B). Samples taken during this revision also remained sterile and postoperative X-rays showed correct implantation of the knee arthrodesis device (Fig. 1C).

I.v. vancomycin was administered for 2 weeks followed by linezolid 600mg p.o. for additional 4 weeks.

After arthrodesis surgery, the further course was uneventful. Blood samples as well as drainage fluid were tested for silver content 24 and 48 hours after surgery. Silver blood concentrations after 48 hours remained under the detection limit of 2 ppb, whereas the silver concentrations in the wound drainage fluid reached 170 and 57 ppb 24 and 48 hours post- operatively, respectively. The patient was followed up regularly in our outpatient clinic for meanwhile 26 months. Full weight-bearing has been possible since inpatient treatment. No silver-specific adverse events such as argyria occurred over the whole follow-up period. No signs or symptoms of recurrent infection were present as well as inflammatory markers continued to remain inconspicuous. Silver serum concentrations remained below the detection limit of 2 ppb. X-rays after 26 months showed stable implant conditions (Fig. 1D).

## Discussion

The new information in this article is the use of a low-amount silver coating technology for an intramedullary knee arthrodesis device and two different aspects that can derived from this case.

The first one is the successful treatment and limb salvage of a recurrent periprosthetic knee joint infection with this low amount metallic silver technology. The described patient showed successful wound healing and no signs of infection recurrence after the implantation of metallic silver coated knee arthrodesis with a total silver amount of only 825 µg after 26 months. All postoperative X-rays were free of signs of septic or aseptic loosening of the intramedullary arthrodesis device. No further revision surgery was required after the arthrodesis. The fact that unsalvageable periprosthetic knee joint infections can be treated with silver-coated knee arthrodesis in this case is in line with another published case series of 8 patients that were successfully treated with another type of silver-coating (MUTARS^®^, Implantcast, Buxtehude, Germany) 2. The authors of this case series concluded that the use of a silver-coated arthrodesis nail is successful in eradicating infection and allowing limb conservation and in should be considered as an alternative to amputation for patients with a recurrent or recalcitrant periprosthetic knee joint infection. Although no detailed information on the total amount of silver on the arthrodesis nails is given from these authors 2, it can be assumed that this is in the range of 0.33 - 2.89 g per implant published for silver-coated MUTARS megaendoprostheses for the treatment of bone tumors 10. This means that the presented low-amount silver technology of this case uses only around 0.2 % to 0.03% of the total amount used for the MUTARS technology.

Another considerable difference between this relatively high silver amount MUTARS and the presented low-amount metallic silver implants is that the intramedullary parts of the arthrodesis nail are not silver-coated for the MUTARS implants but were silver-coated in the current case. One reason for this might be the lack of data demonstrating safe osseointegration of the intramedullary parts of the implant with this high amount silver-concentration.

The reduced silver amount is of interest for the clinical safety of the metallic silver coating technology. The presented patient did not show any adverse events attributable to the silver-coating. Silver serum levels remained under the detection limit of 2 ppb over the entire treatment period of 26 months. This is a different finding compared to the MUTARS technology, in which silver serum concentrations up to around 200 ppb were reported 11. Hardes et al. detected mean silver concentration between 1.93 to 12.98 ppb between the third and 24th postoperative month with a maximum of 56.40ppb 15 months after surgery 10. These elevated blood concentrations were not associated with significant changes in liver and kidney functions measured by laboratory values and were deemed non-toxic.

Argyria, a local blue to bluish grey skin discoloration, was not detected in the presented patient. Argyria was described for the MUTARS technology in up to 23% (7 of 32 patients) after a median of 25.7 months (interquartile range 2 to 44.5 months) in a case series from Glehr et al. (2013) for megaendoprostheses 11. These authors also reported that there was no direct association between the development of argyrosis and blood silver levels. However, it seems logical that reduction of the total silver amount is favorable to improve the clinical safety of silver-coated implants, which is also the intent of the presented low amount metallic silver technology.

## Conclusion

This case report shows the successful treatment of a recurrent periprosthetic knee joint infection with the use a low-amount silver coating technology for an intramedullary knee arthrodesis device. Furthermore, good clinical biocompatibility with silver blood level concentrations below the detection limit of 2 ppb and the absence of argyria was demonstrated.
